# Reduced racial disparity in receipt of optimal locoregional treatment for women with early-stage breast cancer

**DOI:** 10.1371/journal.pone.0291025

**Published:** 2023-09-01

**Authors:** Hasan Nadeem, John A. Romley, Shaneda Warren Andersen

**Affiliations:** 1 Department of Population Health Sciences, School of Medicine and Public Health, University of Wisconsin-Madison, Madison, WI, United States of America; 2 Department of Medicine, University of Washington, Seattle, WA, United States of America; 3 USC Schaeffer Center for Health Policy and Economics, Los Angeles, CA, United States of America; 4 USC School of Pharmacy, Los Angeles, CA, United States of America; 5 USC Price School of Public Policy, Los Angeles, CA, United States of America; 6 University of Wisconsin Carbone Cancer Center, Madison, WI, United States of America; UNT Health Science Center: University of North Texas Health Science Center, UNITED STATES

## Abstract

**Introduction:**

Racial disparities in breast cancer treatment contribute to Black women having the worst breast cancer survival rates in the U.S. We investigated whether differences in receipt of optimal locoregional treatment (OLT), defined as receipt of mastectomy, breast-conserving surgery, or no surgery when contraindicated, existed between Black and White women with early-stage breast cancer from 2008–2018.

**Methods:**

In this retrospective cohort study, data from the Surveillance, Epidemiology, and End Results (SEER) Program Incidence Database was utilized to identify tumor cases from Black and White women aged 20–64 years old with stage I-II breast cancer. Logistic regression analyses were used to evaluate the associations between race and receipt of OLT as well as potential effect modification by tumor characteristics, and year of diagnosis.

**Results:**

Among 177,234 women diagnosed with early-stage breast tumors, disparities in OLT between Black and White women were present from 2008–2010 (2008: 82.1% Black vs. 85.7% White, p<0.001; 2009: 82.1% Black vs. 85.8% White, p<0.001; 2010: 82.2% Black vs. 87.2% White, p<0.001). This disparity was eliminated between 2010–2011 (86.3% Black vs. 87.5% White, p = 0.15), and did not reoccur during the remainder of the study period. From 2010–2011, more Black women received radiation therapy following breast-conserving surgery (43.4% to 48.9%; p = 0.001), which accounted for an overall increased receipt of OLT.

**Conclusion:**

Increased receipt of radiation therapy with breast-conserving surgery appeared to drive a substantial increase in OLT for Black women from 2010–2011 that lasted throughout the study period. Further research on the underlying mechanisms that reduced this disparity is warranted.

## Introduction

Breast cancer is the second leading cause of cancer death in women in the United States, where 43,700 women are expected to die of the disease in 2023 [1]. Currently, the 5-year survival rate for localized breast cancer is over 99% [2]; however, favorable survival statistics are not equitably distributed across race or ethnicity. Black and White women experience similar disease incidence, yet Black women are more likely to die from the disease (28.4 deaths per 100,000 vs. 20.3 per 100,000 for Black and White women, respectively) [3]. The reasons for this differential mortality are multifactorial, some of which include: clustering of more aggressive breast tumor phenotypes in Black women [4, 5], as well as environmental and structural racism that lead to diagnostic delays [6–8]. For instance, studies show that for Black women, place of residence and area-level poverty are related to a delayed diagnosis of breast cancer resulting in increased tumor size and stage at diagnosis [9–11]. However, even among women who have similar tumor biology, stage, and size, disproportionately poor outcomes persist for Black women with breast cancer [12], suggesting that disparate treatment may contribute to adverse outcomes for Black women.

Among women with operable breast tumors, Black women are less likely than White women to receive surgical resection of the tumor by breast-conserving surgery (BCS) or radical mastectomy [13]. Furthermore, it has been shown that Black women are less likely to receive radiation therapy following breast-conserving surgery [14]. Yeboa et al. conducted a Surveillance Epidemiology, and End Results (SEER) study that showed a racial disparity in receipt of radiation therapy following breast-conserving surgery between Black and White women prior to 2010 [15]. Importantly, a more recent study in a single US state (Georgia) from 2012–2016 found no association between receipt of radiation therapy and race which is indicative of signs of improvement in access to radiation therapy following breast-conserving surgery [16]. Nonetheless, it remains unclear whether disparities in first-course of locoregional treatment for breast cancer exist among Black and White women with early-stage tumors at the national level.

The aim of this study was to compare the receipt of optimal locoregional treatment of early-stage breast cancer between Black and White women. We focus on the racial group of Black racial identity because there is a longstanding, persistent racial disparity where Black women have the worst breast cancer survival rates of all racial groups in the US. To investigate this aim, we employed a retrospective cohort design utilizing the National Cancer Institute (NCI) SEER Research Plus Incidence Database to identify Black and White women between the ages 20–64 years old with a diagnosis of operable breast cancer to evaluate the association between Black race and receipt of optimal locoregional therapy.

## Methods

### Study sample

The SEER database, sponsored by the NCI, collects incidence data from eighteen population-based registries that cover approximately 34.6% of the United States population [17]. Data collected by SEER is de-identified prior to public dissemination, thus ethics committee/IRB approval was waived. Similarly, the requirement for informed consent was also waived. We performed a retrospective cohort analysis utilizing the SEER Incidence Research Plus Database to abstract patient cases of 345,988 Black and White women between the ages of 20–64 who were diagnosed with early-stage breast tumors (stage I-II) from 2008–2018. We excluded women over age 64 for multiple reasons. Specifically, women over age 65 are enrolled in Medicare insurance which may influence their treatment decisions. Additionally, several studies published during the study period, including the PRIME II clinical trial [18], showed low utility of radiation therapy following breast-conserving surgery in women 65 years or older, and lastly, Medicare claims data is not available within our dataset. Additional exclusion criteria were applied in a stepwise manner beginning with tumor stage equal to 0 or greater than II (n = 60,292), unknown tumor size or size greater than 2.0 cm (n = 108,080), followed by patient death prior to treatment (n = 34), and lastly a tumor histology consistent with: transitional cell papillomas and carcinomas, adnexal and skin appendage neoplasms, mucoepidermoid neoplasms, nevi or melanoma, soft tissue tumor or sarcoma, fibromatous neoplasm, complex mixed and stromal neoplasms, synovial-like neoplasm, or blood vessel tumors (n = 348). The final sample used for analysis consisted of 177,234 women with early-stage breast cancer.

### Study variables

The outcome variable was optimal locoregional therapy, which was defined using the guidelines developed by the 2019 National Comprehensive Cancer Network (NCCN) Practice Guidelines in Oncology [19]. Optimal locoregional treatment is defined as: as receipt of mastectomy, breast-conserving surgery followed by radiation therapy, or no surgery due to contraindications/comorbidities. Women were defined as having undergone mastectomy if they were coded to have a simple mastectomy, bilateral mastectomy, modified radical mastectomy, radical mastectomy, or extended radical mastectomy. Breast-conserving surgery was defined as women who were coded to have undergone a partial mastectomy (including partial mastectomy with nipple resection, lumpectomy or excisional biopsy, re-excision of the biopsy site, segmental mastectomy, or subcutaneous mastectomy also called nipple-sparing mastectomy). Patients that received beam radiation, radioactive implants, radioisotopes, combined radiotherapy, or radiation not otherwise specified, were defined to have received radiation therapy. Variable codes that met the criteria for receipt of optimal locoregional therapy were surgery that was performed and designated as mastectomy (as defined above), surgery that was performed and designated as breast-conserving surgery (as defined above) concomitant with a code for receipt of radiation therapy, or no surgery because it was not recommended or was contraindicated due to other medical conditions. Non-optimal treatment was defined as individuals who underwent breast-conserving surgery but did not receive radiation therapy thereafter or individuals who did not receive recommended surgery.

Previous iterations of archived guidelines were also reviewed and though there were slight variations in guideline recommendations, such as type of radiation treatment changing to unfractionated from fractionated, the indications for breast cancer surgery remained the same and did not impact the definition of optimal locoregional treatment. We further selected for patients with a discretionary indication for surgery (local therapy) and opted to select for breast tumors <2.0 cm in size such that tumor size would not influence the type of cancer-directed surgery (mastectomy vs. breast-conserving surgery) or introduce a non-surgical first-course of treatment (e.g. neoadjuvant chemo-/radiotherapy) [20]. Histologic subtypes with a non-surgical first-course of treatment (e.g. inflammatory breast cancer or melanoma) or low frequency of counts were excluded from the analysis.

The explanatory variable was a categorial variable for women coded as Black or White race as recorded by the NCI. Cancer registries use various data sources to abstract a coding for race, which includes patient intake forms, medical records, administrative databases, and death certificates [21]. One study comparing SEER records to self-reported Census Bureau survey data shows that concordance is highly sensitive for Black (91%) and White (99%) individuals, while the sensitivity for agreement is much lower for multiracial and American Indian/Alaskan Native (AIAN) groups (<40%) [22].

Demographic variables were age group (>19 and <45, 45–54, and 55–64) and marital status coded by single, married, separated, divorced, widowed, unmarried, or unknown. The SEER Incidence Research Plus database utilized several variables to define breast tumor stage depending on the year of diagnosis: the American Joint Committee on Cancer (AJCC) 6^th^ edition staging for cases prior to 2016, the derived SEER Combined Stage Group for breast cancer cases diagnosed in 2016 and 2017, and the Extent of Disease (EOD) summary stage criteria for breast cancer cases diagnosed in 2018. Tumor stage variables were created for consistency and to identify tumors that fell into the stage categories: IA, IB, IIA, and IIB.

Regarding tumor biology characteristics, the variables utilized in the analysis were tumor size, subtype, grade, and histology. Tumor size categories were 0–1.0 cm and 1.1–2.0 cm. Tumor subtype data was not available for cases prior to 2010. For cases after 2010, tumor subtype is coded by HER2 status (positive, negative, or unknown) and Hormone Receptor (HR) status (ER positive, negative, or unknown and/or PR positive, negative, or unknown). Tumor grade is classified as I-IV (well-differentiated, moderately-differentiated, poorly-differentiated, and undifferentiated). In the year 2018, the SEER Incidence database introduced the clinical grade and pathologic grade variables. The former is a recording of tumor grade determined prior to treatment and was utilized in our study for breast tumors diagnosed in 2018. Finally, our study incorporated a broad range of breast tumor histologies (with the exception of those mentioned in the exclusion criteria above) and the full list of histologies documented within the SEER 2020 Research Plus Incidence dataset can be referenced in the November 2020 submission data description [23]. For brevity, the histologic subtypes in this analysis were classified as ductal/lobular or non-ductal/lobular.

### Statistical analysis

A descriptive statistical analysis on sociodemographic, tumor biology, and treatment factors was performed. All statistical T-tests were two-tailed and evaluated at a significance level of 0.05. Multivariable logistic regression was used to evaluate the relationship between race and receipt of optimal locoregional therapy. The outcome variable is defined by receipt of mastectomy, breast-conserving surgery with radiation therapy, or no surgery due to contraindications. It is coded as a binary (yes/no) variable in which no would be breast-conserving surgery without radiation therapy or no surgery without contraindications. The primary exposure variable was Black or White race. We performed univariate regression analyses for each control covariate (e.g. age group, tumor size, etc.) that was included in the full model as well as the explanatory variable (race) on the outcome variable (receipt of optimal locoregional therapy). The univariate analysis output is displayed alongside the full multivariate regression output in the **S1 Table**.

The overall model included adjustment with patient sociodemographic covariates (age group at diagnosis and marital status) as well as tumor characteristic covariates (tumor stage, size, grade, and histology). Prior to 2010, a persistent disparity was observed between Black and White individual’s receipt of radiation therapy following breast-conserving surgery [15]. However, an updated study (2012–2016) on women undergoing breast cancer surgery in Georgia showed improvements in access to radiation therapy [16]. With the background information, we hypothesized that a national increase in access to radiation therapy due to secular changes such as the passing of the Affordable Care Act might differentially impact Black and White women. For this reason, we tested the multiplicative interaction term between race and year of diagnosis, which allowed for a more granular assessment on yearly changes in receipt of optimal locoregional treatment for Black women compared to White women. The tumor subtype variable was not recorded for cases prior to 2010 and included in statistical models when available. All statistical analyses were completed using Stata, version 17.0.

## Results

### Descriptive characteristics

Our sample included 177,234 women that were diagnosed with early-stage breast cancer. The study sample was 88.61% White, 63.00% married, and 50.28% within the age group of 55–64 years old (***Table 1***). White women were diagnosed with breast cancer at an older age compared to Black women. There were fewer Black women diagnosed with early-stage breast tumors in the age group of 55–64 years of age (48.22% Black vs. 50.55% White, p<0.001), yet more Black women with breast tumors in the age group of 20–44 years of age (16.42% Black vs. 13.67% White, p<0.001).

**Table 1:** Descriptive characteristics stratified by race, SEER data 2008–2018.

Characteristic	All (N = 177,234)	Black (N = 20,189)	White (N = 157,045)	p-value
**Age at Diagnosis (%)**				
20–44	13.99	16.42	13.67	<0.001
45–54	35.73	35.36	35.78	0.244
55–64	50.28	48.22	50.55	<0.001
**Marital Status (%)**				
Single	15.96	32.80	13.80	<0.001
Married	63.00	40.44	65.90	<0.001
Separated, Divorced, or Widowed	16.00	20.98	15.36	<0.001
Unmarried or Domestic Partner	0.39	0.24	0.41	<0.001
Unknown	4.65	5.54	4.53	<0.001
**Stage (%)**				
IA	82.75	78.80	83.26	<0.001
IB	1.70	2.52	1.59	<0.001
IIA	15.38	18.41	14.99	<0.001
IIB	0.17	0.27	0.16	<0.001
**Breast Cancer Subtype (%)**				
HR+/HER2+	7.84	9.28	7.66	<0.001
HR-/HER2+	2.65	3.59	2.53	<0.001
HR+/HER2-	60.62	52.99	61.60	<0.001
Triple Negative	6.95	13.35	6.13	<0.001
Unknown Subtype*	3.64	4.20	3.80	0.005
**Tumor Size (%)**				
0.1–1.0cm	42.92	39.65	43.34	<0.001
1.1–2.0cm	57.08	60.35	56.66	<0.001
**Grade (%)**				
I	29.21	20.08	30.39	<0.001
II	43.04	39.46	43.50	<0.001
III	23.71	35.60	22.18	<0.001
IV	0.24	0.30	0.24	0.071
Undetermined	3.15	3.95	3.05	<0.001
**Histology (%)**				
Ductal or Lobular	96.04	95.60	96.10	<0.001
Non-Ductal or -Lobular	3.96	4.40	3.91	<0.001
**Treatment (%)**				
No Surgery	6.13	10.22	5.61	<0.001
BCS	64.53	63.12	64.72	<0.001
BCS + RT	50.88	48.45	51.19	<0.001
Mastectomy	33.62	33.86	33.59	0.459
OLT	85.60	84.17	85.78	<0.001

HR = Hormone Receptor; BCS = Breast-Conserving Surgery; RT = Radiation Therapy; OLT = Optimal locoregional therapy

*Unknown HR or Her-2 status for tumors from 2010–2018

### Tumor stage & biology

Most women in our sample were diagnosed with stage IA breast cancer (82.75%), followed by stage IIA (15.38%). Most tumor samples exhibited the following tumor biology: a tumor subtype that is HR-positive and HER2-negative (60.62%), a tumor size between 1.1–2.0cm (57.08%), tumor grade II (43.04%), and ductal or lobular tumor histology (96.04%).

**Fig 1:**
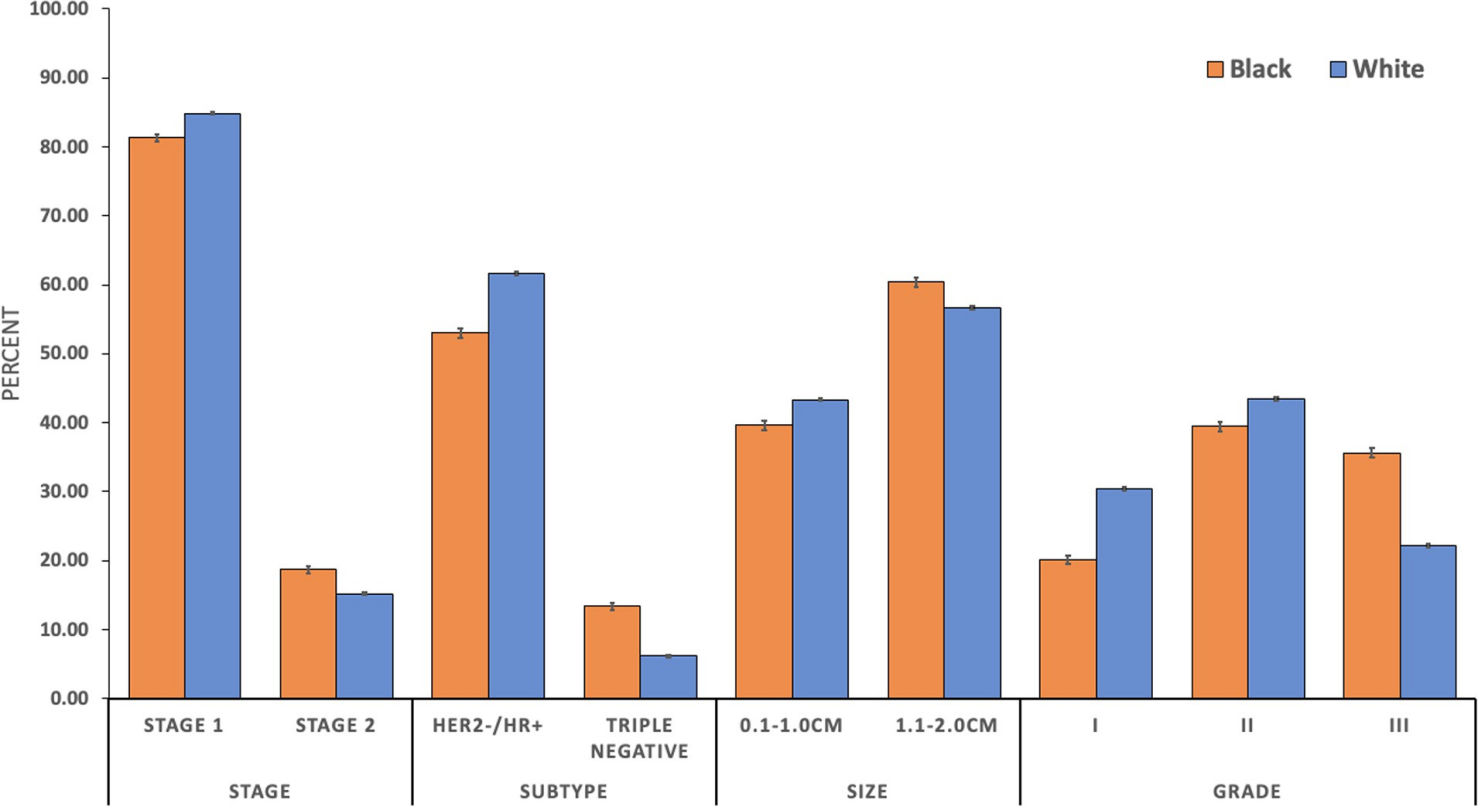
Clinicopathologic characteristics for black and white women. Stage 1 = Stage IA & Stage IB; Stage 2 = Stage IIA & Stage IIB p-value < 0.001 for all displayed tumor characteristics between Black and White women.

Tumor characteristics varied by race. Black women were less likely to be diagnosed with stage IA tumors (78.80% Black vs. 83.26% White, p<0.001) (***Fig 1***). Additionally, the triple negative tumor subtype (i.e. HR-negative and HER2-negative) was more common in Black women (13.35% Black vs. 6.13% White, p<0.001). Among tumors <2.0cm, Black women were more likely to be diagnosed with a tumor larger than 1.0cm (60.35% Black vs. 56.66% White, p<0.001). Regarding tumor histopathology, Black women were more often diagnosed with grade III tumors (35.60% Black vs. 22.18% White, p<0.001) and a histological subtype other than lobular or ductal (4.40% Black vs. 3.91% White, p<0.001).

### Treatment

While breast-conserving surgery was the more common treatment option compared to mastectomy (64.53% vs. 33.62%, p<0.001), Black women received less breast-conserving surgery than White women (63.12% vs. 64.72%, p<0.001) over the study period. The rates of mastectomy were similar for both races (33.86% vs. 33.59%, p = 0.446). Black women experienced a 3.5% decrease in receipt of mastectomy from 2008 to 2018 and White women experienced a 7.8% decrease over the same period.

**Fig 2:**
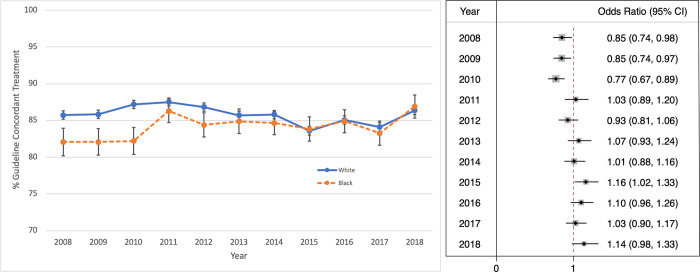
**A.** Receipt of optimal locoregional therapy stratified by race and year of diagnosis. **B.** Odds Ratio of Black Patients Receiving Optimal Locoregional Therapy by Year Relative to White Patients.

The total receipt of optimal locoregional therapy on average was high (85.60%) (***Fig 2A***). From 2008 to 2018, Black women experienced a 4.21% increase in receipt of optimal locoregional therapy, larger than the 0.63% increase in receipt of optimal locoregional therapy experienced by White women.

Between 2008–2010, there was a disparity in receipt of optimal locoregional therapy between Black and White women. Receipt of optimal locoregional therapy for Black women was significantly less compared to White women in 2008 (82.07% Black vs. 85.73% White, p<0.001), 2009 (82.07% Black vs. 85.84% White, p<0.001), and 2010 (82.21% Black vs. 87.18% White, p<0.001). Between 2010 and 2011, Black women experienced a substantial increase in receipt of optimal locoregional therapy from 82.21% to 86.29%, which eliminated the treatment disparity between Black and White women (86.29% Black vs. 87.51% White, p = 0.1473). No difference was observed during the remainder of the study period.

**Fig 3:**
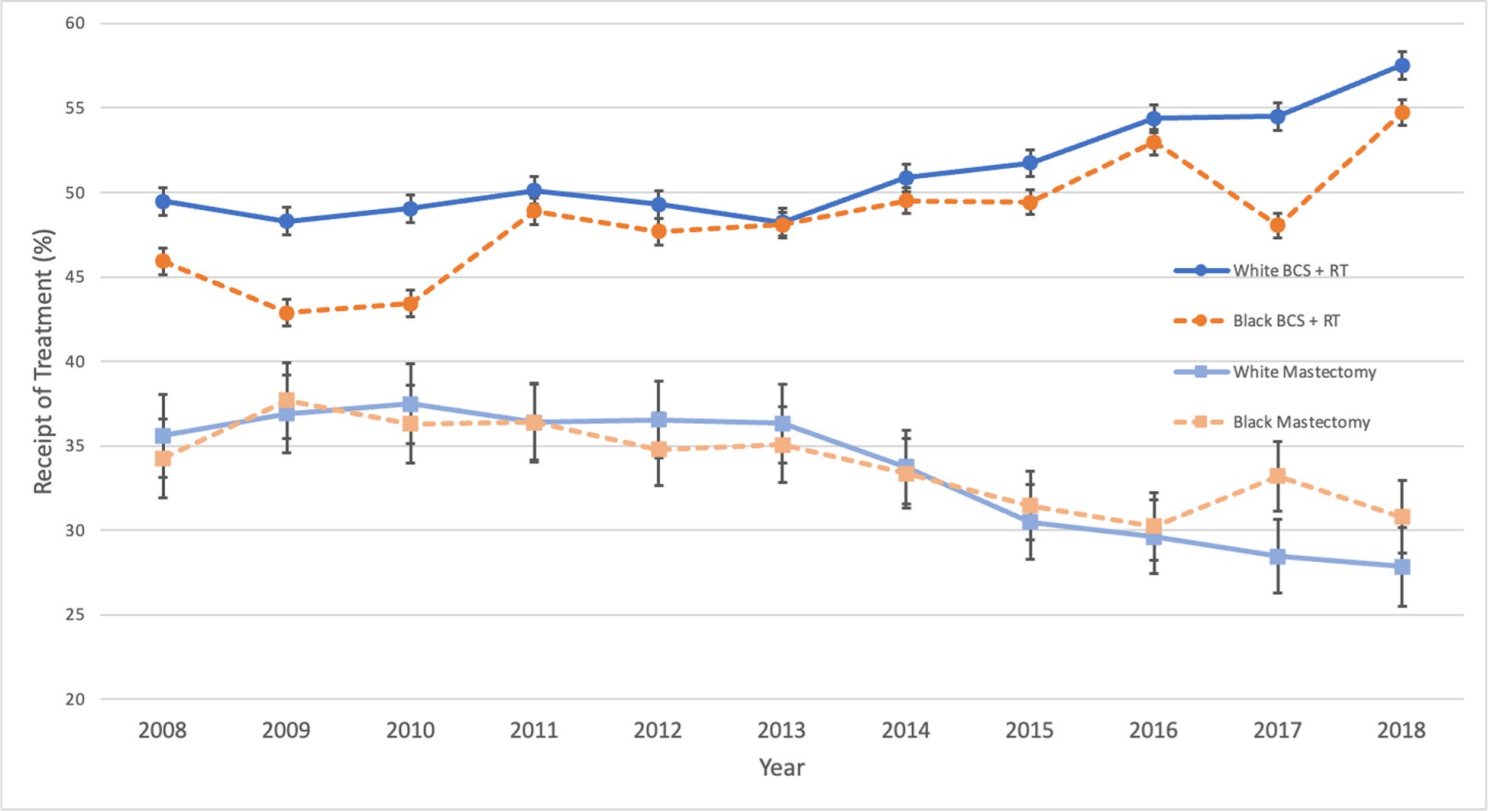
Receipt of mastectomy vs. breast conserving surgery (breast-conserving surgery) + radiation therapy (radiation therapy) stratified by race and year of diagnosis.

Receipt of breast-conserving surgery with radiation therapy was a contributing factor for the initial racial disparity in receipt of optimal locoregional therapy (***Fig 3***). Prior to 2011, the receipt of breast-conserving surgery with radiation therapy was less than 46% for Black women whereas the receipt of breast-conserving surgery with radiation therapy for White women was greater than 48%. Between the years 2010 and 2011, the rate of breast-conserving surgery with radiation therapy increased substantially for Black women from 43.4% to 48.9%, which resulted in an equalization of receipt of breast-conserving surgery with radiation therapy between Black and White women starting in 2011 (48.9% Black vs. 50.1% White, p = 0.34). The increased receipt of breast-conserving surgery with radiation therapy after 2010 persisted throughout subsequent years and established a new floor for receipt of breast-conserving surgery with radiation therapy for Black women.

Black women exhibited a decrease in receipt of breast-conserving surgery with radiation therapy in 2017 (48.0%), which was accompanied by an increase in mastectomy for Black women during that same year (33.2%). While the decreased receipt of breast-conserving surgery with radiation therapy and increased receipt of mastectomy slightly increased the gap in receipt of optimal locoregional therapy between Black and White women, the magnitude of this effect did not reproduce the disparity between races (p = 0.33).

### Multivariable regression analysis

Over the study period, Black race was associated with a lower likelihood of receiving optimal locoregional therapy (OR = 0.85; 95% CI: 0.74–0.98; p = 0.02) (***Table 2***). Predictive margins yield a point estimate of the conditional predicted mean of receipt of optimal locoregional therapy when covariates are held at fixed values. This is interpreted as the percent likelihood an individual of Black or White race would receive optimal locoregional therapy based upon the full multivariable regression model. Over the study period, the point estimate for receipt of optimal locoregional therapy for Black women is 85.46% (95% CI 84.98–85.94, p<0.001), compared to 85.61% for White women (95% CI 85.43–85.78, p<0.001).

**Table 2:** Associations between selected patient characteristics and optimal locoregional therapy.

Variable^a^	OR (95% CI)	p-value
**Race**		
White	Ref	-
Black	0.85 (0.74–0.98)	0.021
**Year of Diagnosis**		
2008 (n = 15,803)	Ref	-
2009 (n = 16,258)	1.01 (0.94–1.08)	0.787
2010 (n = 15,589)	1.13 (1.05–1.21)	0.001
2011 (n = 16,116)	1.16 (1.09–1.25)	<0.001
2012 (n = 16,164)	1.09 (1.02–1.17)	0.010
2013 (n = 16,194)	0.99 (0.93–1.06)	0.853
2014 (n = 16,450)	1.00 (0.93–1.07)	0.944
2015 (n = 16,600)	0.84 (0.78–0.89)	<0.001
2016 (n = 16,391)	0.92 (0.86–0.98)	0.018
2017 (n = 16,365)	0.86 (0.80–0.91)	<0.001
2018 (n = 15,341)	1.00 (0.93–1.07)	0.940
**Tumor Size**		
Size < 1.0cm	Ref	-
Size < 2.0cm	0.90 (0.88–0.93)	<0.001
**Stage**		
Stage IA	Ref	-
Stage IB	1.10 (0.98–1.23)	0.080
Stage IIA	0.91 (0.88–0.95)	<0.001
Stage IIB	1.41 (1.00–2.00)	0.050
Interaction: Race*Year of Diagnosis ^b^		
Black*2008	Ref	-
Black*2009	0.99 (0.82–1.20)	0.932
Black*2010	0.90 (0.75–1.10)	0.309
Black*2011	1.20 (0.98–1.46)	0.079
Black*2012	1.08 (0.89–1.30)	0.454
Black*2013	1.22 (1.01–1.49)	0.042
Black*2014	1.21 (1.00–1.47)	0.046
Black*2015	1.35 (1.12–1.63)	0.002
Black*2016	1.30 (1.08–1.58)	0.006
Black*2017	1.26 (1.04–1.52)	0.016
Black*2018	1.37 (1.12–1.67)	0.002

OR = Odds Ratio; HR = Hormone Receptor; Triple Negative = HR—& HER2 -

To preserve brevity, select covariate output is displayed. Full multivariate regression output on OLT is shown in the S1 Table.

^a^The adjusted statistical model includes variables for race, year of diagnosis, marital status, age, tumor grade, tumor size, tumor stage, tumor histology, and an interaction term for race *interacted with* year of diagnosis

When stratifying the multivariable regression analysis prior to vs. after the year 2011, we observe broader differences between the point estimates for Black and White womens’ receipt of optimal locoregional therapy. From 2008–2010, the point estimate for optimal locoregional therapy for Black women was 83.73% (95% CI: 82.73–84.73, p<0.001), compared to 86.07% for White women (95% CI: 85.74–86.40, p<0.001). From 2011–2018, the point estimate for Black women’s receipt of optimal locoregional therapy was 86.24% (95% CI: 85.70–86.78, p<0.001), which eclipses the estimation of 85.41% (95% CI: 85.21–85.62, p<0.001) for White women.

Our analysis showed a diminished racial difference in receipt of optimal locoregional therapy after 2010 (***Fig 2****B****)***. Between 2010 and 2011, the OR associated with receipt of optimal locoregional therapy for Black women changed from 0.77 (95% CI: 0.67–0.89) to 1.03 (95% CI: 0.89–1.20).

## Discussion

### Principal findings

This study finds an increased receipt of optimal locoregional therapy for early-stage breast tumors among Black women that is attributable to an increase in receipt of radiation therapy following breast-conserving surgery.

### Interpretation of Results

We place our results within the context of previous studies that have investigated potential causes of the adverse breast cancer outcomes experienced by Black Americans. We and others show Black women are diagnosed at a younger age with larger, more advanced and more aggressive tumors [3, 13, 24, 25]. Additionally, Black women are more often of lower socioeconomic status, measured in the present study by marital status, which is also linked to higher breast cancer mortality [26–29]. Differences in tumor characteristics, socioeconomic status, and age at diagnosis suggest that Black women may continue to experience higher breast cancer mortality even as the racial disparity in optimal locoregional therapy is reduced.

Additionally, these differences in patient-level and tumor-level characteristics may influence breast cancer treatment decisions [30–33].

### Limitations and strengths

The present study has many notable strengths and weaknesses. Strengths of this study include the cohort design, and the timeliness of the analysis. Additionally, the SEER database is a large, nationally representative population-based patient sample. To ensure the disparity in optimal locoregional therapy was not a temporary trend, we performed sensitivity analyses that extended from the beginning of the study period and identified that the disparity in receipt of optimal locoregional therapy between Black and White women existed prior to 2008 as well. We also reincorporated the variable of insurance status (which was no longer recorded after 2016) and found that this did not affect our results from 2008–2016.

However, the study has specific limitations, including that SEER notes that collection of radiation therapy data is incomplete. However, analysis from one study evaluating the concordance between SEER data and SEER-Medicare data reported that SEER treatment data has a high positive predictive value and a sensitivity for radiation therapy greater than 80% [34]. Furthermore, this study showed that the positive predictive value and sensitivity of SEER radiation therapy did not vary greatly by race or ethnicity [34]. Another study found that overall agreement with SEER radiation therapy data for breast cancer and a self-reported patient survey to be 83% [35]. These studies suggest that our data may underestimate the receipt of radiation therapy following breast-conserving surgery, and thereby, receipt of optimal locoregional therapy.

Additionally, we did not have access to data on individual- or area-level socioeconomic status. Previous research has demonstrated that the effect of racial disparities in cancer care is reduced, but persists, when controlling for social and economic factors [36]. Our analysis did account for marital status which may be used as a marker of socioeconomic status and the data suggests that Black women were more often of single marital status which is linked to higher breast cancer mortality [26–29]. Additionally, to preserve patient privacy, various identifiers were removed from SEER Incidence data such as geographic location and the type of hospital the patient received their care. This information would have allowed us to control for factors such as hospital teaching status, health system and local health insurance coverage, supply of radiation oncologists, and surgical volume, which have been shown to affect receipt of breast cancer surgery and radiation therapy [37]. Incorporation of patient-level and physician-level factors would have been useful in this analysis. It has been established that factors such as distance to hospital or treatment facilities, use of private vs. public transportation, and insurance status, can all impact receipt of optimal locoregional therapy in women with early stage breast tumors [36, 38–40].

Due to lack of data availability, other racial groups beyond Black and White Americans were omitted from the analysis, including the exclusion of American Indian/Alaskan Native (AIAN) individuals who also experience breast cancer survival disparities. We also did not consider the differential effects of ethnicity by race on receipt of optimal treatment. The lack of data partially reflects the persistent, structural barriers that stymie representation in healthcare data.

Another group excluded from our analysis are women aged 65 and older. Several studies were published during the study period that supported the omission of radiation therapy following breast-conserving surgery for women over 65 years old [18, 41, 42]. The role of radiation therapy in older women with early-stage breast cancer remains a topic of discussion. By restricting our population to less than 65 years of age, we ensured a study population better suited to assess changes in receipt of optimal locoregional treatment for women with operable breast tumors. Furthermore, most women over 65 are insured by Medicare, and Medicare/Medicaid status has been shown to influence breast cancer surgery treatment decisions [43–45]. We did not have access to Medicare data in the Research Plus dataset and could not fully explore how changes in insurance status might impact receipt of optimal locoregional treatment. For these reasons, we felt compelled to assess a study population less than 65 years of age.

Finally, we acknowledge that our study findings are not generalizable to the sub-populations of women with an advanced stage at diagnosis or a tumor subtype that would warrant systemic treatment of disease. Persistent disparities have been documented in the use of endocrine therapy and chemotherapy for treatment of early-stage as well as metastatic breast cancer [46–49]. Efforts in making a more equitable treatment landscape for breast cancer should focus on locoregional treatment, and systemic treatment of the disease.

Our study assesses a broad contingent of women affected by breast cancer and is generalizable to Black and White women under the age of 65 with stage I or stage II disease that meet indications for operative therapy. Notably, there are important parts of the total breast cancer population who are not represented in this analysis.

### Implications and conclusions

This study builds upon the research conducted by Yeboa et al. and Rothley and adds to the knowledge base by providing a broader view of the receipt of optimal locoregional therapy among Black and White women with early-stage breast tumors. The novel finding in this study is that there are similar rates of receipt of local optimal locoregional therapy between Black and White women following the year 2010, which appears to be driven by an increase in receipt of radiation therapy following breast-conserving surgery for Black women. Implementation of the Affordable Care Act occurred in 2010 and may have contributed to increased receipt of optimal locoregional treatment. Future studies with more granular-level information on insurance type and individual-level covariates will be better suited to assess how this important change in the healthcare landscape has influenced breast cancer treatment.

The reduced racial disparity in receipt of radiation therapy for breast cancer treatment is a welcome sign that disparities in cancer care may be reduced, but sustained effort will be required to maintain these findings [50]. Importantly, unadjusted rates show that Black women consistently received less breast-conserving surgery and radiation therapy than White women. Identifying the specific factors that drive receipt of radiation therapy following breast-conserving surgery across race may inform interventions that address racial disparities within the breast cancer treatment paradigm.

## Supporting information

S1 Checklist (DOCX)

S1 Table Univariate and multivariate logistic regression analyses with optimal locoregional therapy as outcome variable HR = Hormone Receptor; BCS = breast-conserving surgery; RT = radiation therapy; OLT = optimal locoregional therapy *Unknown HR or Her-2 status for tumors from 2010–2018. (DOCX)
