# Supplementary material for: Reduced racial disparity in receipt of optimal locoregional treatment for women with early-stage breast cancer
Source: PLoS One. 2023 Sep 1;18(9):e0291025. doi: 10.1371/journal.pone.0291025 (PMC10473527; doi:10.1371/journal.pone.0291025)
Supplement: S1 Table — (DOCX) [file pone.0291025.s002.docx]

| **S1 Table.** Univariate and Multivariate Logistic Regression Analyses with Optimal Locoregional Therapy as Outcome Variable | | | | | |
| --- | --- | --- | --- | --- | --- |
|  | **Univariate Analysis** | |  | **Multivariate Analysis** | |
| **Variable** | **OR** | **p-value** |  | **OR** | **p-value** |
| **Race** | | | | | |
| White | Ref | - |  | Ref | - |
| Black | 0.88 | < 0.001 |  | 0.85 | 0.021 |
|  |  |  |  |  |  |
| **Age Group at Diagnosis** | | | | | |
| Age Group | 1.04 | <0.001 |  | 1.02 | <0.001 |
|  |  |  |  |  |  |
| **Marital Status** | | | | | |
| Married | Ref | - |  | Ref | - |
| Single | 0.76 | <0.001 |  | 0.74 | <0.001 |
| Separated, Divorced, or Widowed | 1.00 | 0.93 |  | 0.90 | <0.001 |
| Unmarried | 1.02 | 0.86 |  | 0.96 | 0.701 |
| Unknown Relationship | 0.68 | <0.001 |  | 0.63 | <0.001 |
|  |  |  |  |  |  |
| **Grade** | | | | | |
| I | Ref | - |  | Ref | - |
| II | 1.11 | <0.001 |  | 0.97 | 0.063 |
| III | 0.78 | <0.001 |  | 0.78 | <0.001 |
| IV | 0.76 | 0.029 |  | 0.70 | 0.005 |
| Unknown | 0.73 | <0.001 |  | 0.02 | <0.001 |
|  |  |  |  |  |  |
| **Tumor Size** | | | | | |
| Size < 1.0cm | Ref | - |  | Ref | - |
| Size < 2.0cm | 0.87 | <0.001 |  | 0.90 | <0.001 |
|  |  |  |  |  |  |
| **Stage** | | | | | |
| Stage IA | Ref | - |  | Ref | - |
| Stage IB | 1.03 | 0.561 |  | 1.10 | 0.080 |
| Stage IIA | 0.87 | <0.001 |  | 0.91 | <0.001 |
| Stage IIB | 1.19 | 0.318 |  | 1.41 | 0.050 |
|  |  |  |  |  |  |
| **Histology** | | | | | |
| Ductal/Lobular | Ref | - |  | Ref | - |
| Non-Ductal/Lobular | 0.81 | <0.001 |  | 0.80 | <0.001 |
|  |  |  |  |  |  |
| **Subtype** | | | | | |
| HR+/HER2- | Ref | - |  | - | - |
| HR +/HER2+ | 0.81 | <0.001 |  | - | - |
| HR-/HER2+ | 0.84 | <0.001 |  | - | - |
| Triple Negative | 0.75 | <0.001 |  | - | - |
| Unknown Subtype ^a^ | 0.60 | <0.001 |  | - | - |
|  |  |  |  |  |  |
| **Year of Diagnosis** | | | | | |
| 2008 |  |  |  | Ref | - |
| 2009 |  |  |  | 1.01 | 0.787 |
| 2010 |  |  |  | 1.13 | 0.001 |
| 2011 |  |  |  | 1.17 | <0.001 |
| 2012 |  |  |  | 1.09 | 0.010 |
| 2013 |  |  |  | 0.99 | 0.853 |
| 2014 |  |  |  | 1.00 | 0.944 |
| 2015 |  |  |  | 0.84 | <0.001 |
| 2016 |  |  |  | 0.92 | 0.018 |
| 2017 |  |  |  | 0.86 | <0.001 |
| 2018 |  |  |  | 1.00 | 0.940 |
|  |  |  |  |  |  |
| **Interaction** | | | | | |
| Black * 2008 |  |  |  | Ref | - |
| Black * 2009 |  |  |  | 0.99 | 0.932 |
| Black * 2010 |  |  |  | 0.90 | 0.309 |
| Black * 2011 |  |  |  | 1.20 | 0.079 |
| Black * 2012 |  |  |  | 1.08 | 0.454 |
| Black * 2013 |  |  |  | 1.22 | 0.042 |
| Black * 2014 |  |  |  | 1.21 | 0.046 |
| Black * 2015 |  |  |  | 1.35 | 0.002 |
| Black * 2016 |  |  |  | 1.30 | 0.006 |
| Black * 2017 |  |  |  | 1.26 | 0.016 |
| Black * 2018 |  |  |  | 1.37 | 0.002 |

HR=Hormone Receptor; BCS=Breast-Conserving Surgery; RT=Radiation Therapy; OLT=Optimal locoregional therapy

^a^ Unknown HR or Her-2 status for tumors from 2010-2018
